# Nucleolar Accumulation of RNA Binding Proteins Induced by ActinomycinD Is Functional in *Trypanosoma cruzi* and *Leishmania mexicana* but Not in *T. brucei*

**DOI:** 10.1371/journal.pone.0024184

**Published:** 2011-08-31

**Authors:** Ezequiel Názer, Daniel O. Sánchez

**Affiliations:** Instituto de Investigaciones Biotecnológicas-Instituto Tecnológico Chascomús, UNSAM-CONICET, San Martín, Provincia de Buenos Aires, Argentina; University of Georgia, United States of America

## Abstract

We have recently shown in *T. cruzi* that a group of RNA Binding Proteins (RBPs), involved in mRNA metabolism, are accumulated into the nucleolus in response to Actinomycin D (ActD) treatment. In this work, we have extended our analysis to other members of the trypanosomatid lineage. In agreement with our previous study, the mechanism seems to be conserved in *L. mexicana*, since both endogenous RBPs and a transgenic RBP were relocalized to the nucleolus in parasites exposed to ActD. In contrast, in *T. brucei*, neither endogenous RBPs (TbRRM1 and TbPABP2) nor a transgenic RBP from *T. cruzi* were accumulated into the nucleolus under such treatment. Interestingly, when a transgenic TbRRM1was expressed in *T. cruzi* and the parasites exposed to ActD, TbRRM1 relocated to the nucleolus, suggesting that it contains the necessary sequence elements to be targeted to the nucleolus. Together, both experiments demonstrate that the mechanism behind nucleolar localization of RBPs, which is present in *T. cruzi* and *L. mexicana*, is not functional in *T. brucei*, suggesting that it has been lost or retained differentially during the evolution of the trypanosomatid lineage.

## Introduction

Trypanosomes are protozoan parasites with sanitary relevance, since many members of this group of parasites are causative agents of important and neglected human diseases, such as Chagas disease in America and Sleeping sickness disease in Africa [1]. In addition to their potential impact in human health, trypanosomes are attractive model organisms to study fundamental processes such as RNA metabolism and processing. For instance, in contrast to most eukaryotes, trypanosomes do not regulate mRNA synthesis at the transcriptional level [2], [3]. Gene expression regulation in these organisms is mostly achieved post-transcriptionally by controlling mRNA stability and translation [2], [4].

Trypanosomes are also characterized by having complex life cycles alternating between vertebrate and invertebrate hosts, where they are exposed to different stress conditions that change abruptly [5], [6]; therefore, several and rapid modifications at the gene expression level must be accomplished in order to readapt to such different conditions and niches [7]. In this regard, the rapid formation of stress granules in response to starvation and severe heat shock [8], [9], as well as the relocalization of certain of RNA Binding Proteins (RBPs) and poly(A)+ RNA to the nucleolus induced by particular stress conditions [10], might add another layer of rapid post-transcriptional regulation in these organisms.

The nucleolus is a subnuclear structure which has been traditionally seen as the ribosomes “factory”. More recently, it has been shown that it also plays additional functions related to other cellular processes [11]. Among its novel functions, it has been proposed that it might act as a sensor and coordinator of the stress response [12], [13]. In this respect, there is a growing number of reports showing that key factors are sequestered in the nucleolus during certain stress conditions [14]–[19]. Interestingly, both the *Arabidopsis* and human nucleolar proteomes have shown the unexpected nucleolar localization of RBPs involved in different steps of mRNA metabolism [20]–[22]. In *T. cruzi*, we have recently shown that some RBPs are accumulated into the nucleolus in response to Actinomycin D (ActD) treatment, suggesting a novel potential role of the trypanosome nucleolus in gene expression regulation mechanisms [10]. In this regard, an interesting possibility might be that the nucleolus, in response to certain stress conditions, could sequester RBPs involved in mRNA metabolism in order to modulate the gene expression repertoire.

The aim of this work was to evaluate whether such nucleolar accumulation of RBPs is also functional in other members of the trypanosomatid family. In agreement with our previous results in *T. cruzi*, this mechanism is also conserved in *L. mexicana*. In contrast, in *T. brucei*, neither endogenous RBPs nor a transgenic RBP from *T. cruzi* were relocated into the nucleolus in response to ActD. Together, our results suggest that the mechanism behind the nucleolar relocalization of RBPs in trypanosomes seems to be lost or retained differentially during the evolution of the trypanosomatid lineage.

## Results

### Behaviour of RBPs in *L. mexicana* and *T. brucei* in response to ActD treatment

**Figure 1:**
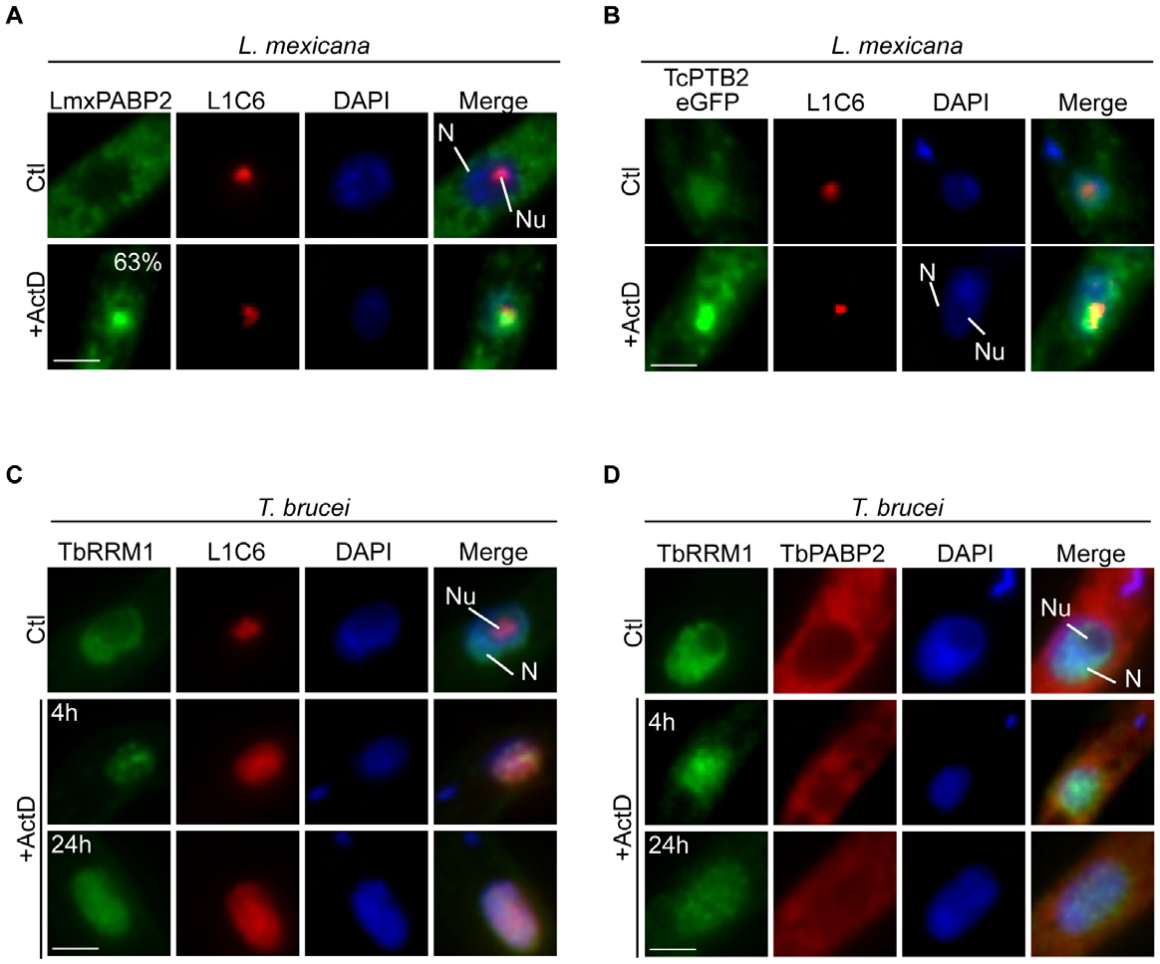
Behaviour of RBPs in *L. mexicana* and *T. brucei* in response to ActD treatment. (A) Immunofluorescence images for endogenous LmxPABP2 in *L. mexicana* in control or ActD-treated parasites for 24 h. LmxPABP2 (green) was colocalized with the nucleolar marker L1C6 (red) and DAPI. (B) Images of TcPTB2-eGFP (green), DAPI and L1C6 are shown in ActD-treated (24 h) and untreated parasites. (C) Immunofluorescence images for endogenous TbRRM1 and (D) TbPABP2 in *T. brucei* in control, after 4 h or 24 h of ActD treatment. TbRRM1 (green) was colocalized with the nucleolar marker L1C6 (red) and DAPI, whereas TbPABP2 (green) was colocalized with TbRRM1 (red) and DAPI. Nuclei were counterstained with DAPI (blue). The forth column on the right is an overlap of each protein analyzed and DAPI. The quantification for LmxPABP2 is expressed as the mean from at least three independent experiments. N: nucleus, Nu: nucleolus. Size bars represent 2 µm. Representative nuclei are shown.

To know whether the mechanism responsible for the nucleolar relocalization of RBPs induced by ActD in *T. cruzi* was also conserved in other trypanosomatids, we evaluated the behaviour of the RBP LmxPABP2 (LmxM.34.4130) in *L. mexicana* promastigotes. The Poly(A)-Binding Protein (PABP) of eukaryotes is a cytoplasmic RBP implicated in different steps of mRNA metabolism [23], [24]. Under normal conditions, LmxPABP2 was exclusively located in the cytoplasm (Figure 1A and S1A, top panels). However, when parasites were subjected to ActD, a transcriptional inhibitor which has extensively been used in several organisms, including trypanosomatids [25], [26], for 24 h, LmxPABP2 was accumulated into the nucleolus in 63% of parasites, since it colocalized with the weakest area of staining with the DNA-specific dye DAPI and with the nucleolar antigen L1C6. It should be mentioned that in most of the parasite population (around 90%), the L1C6 marker was dispersed from the nucleolus to the nucleoplasm after ActD treatment. Therefore, as previously done for *T. cruzi* [10], we used the remaining parasites for colocalization studies. This result is in agreement with the behaviour of the PABP2 orthologue in *T. cruzi* (TcPABP2) [10], suggesting that the mechanism of RBP nucleolar relocalization is also present in *L. mexicana*. To further support this conclusion, we expressed the *T. cruzi* RBP TcPTB2 (Tc00.1047053511727.160), as a C-terminal eGFP fusion protein, in *L. mexicana*. In concordance with its behaviour in *T. cruzi* [10], the TcPTB2 transgenic protein was also accumulated into the nucleolus in response to ActD treatment in a *L. mexicana* context (Figure 1B, bottom panels).

We then extended our study to *T. brucei* procyclic forms, by exploring the behaviour of two RBPs, namely TbRRM1 (Tb927.2.4710) [27] and TbPABP2 (Tb09.211.2150) [28]. Under normal conditions, TbRRM1 was localized throughout the nucleoplasm, presenting a speckled pattern (Figure 1C and S1B, top panels). On the other hand, TbPABP2 exhibited a predominantly cytoplasmic distribution (Figure 1D and S1B, top panels). Both results are in agreement with previous reports [8], [27]. When parasites were treated with ActD for 4 h, the nucleolar marker became dispersed throughout the nucleoplasm in most cells, as previously shown in *T. cruzi* [10]. Interestingly, TbRRM1 remained in speckles, but being these larger and more rounded, whereas TbPABP2 remained in the cytoplasm (Figure 1C and 1D middle panels, and S1B). As this result was quite unexpected, we repeated the experiment treating the parasites for 24 h, and obtained a similar pattern (Figure 1C and 1D, bottom panels).

These results suggest that the mechanism involved in the nucleolar accumulation of RBPs in response to ActD described in *T. cruzi* [10] is also conserved in *L. mexicana*, but might be absent in *T. brucei*.

### Transgene expression analysis demonstrated that the pathway/mechanism involved in nucleolar relocalization of RBPs is absent in *T. brucei*

**Figure 2:**
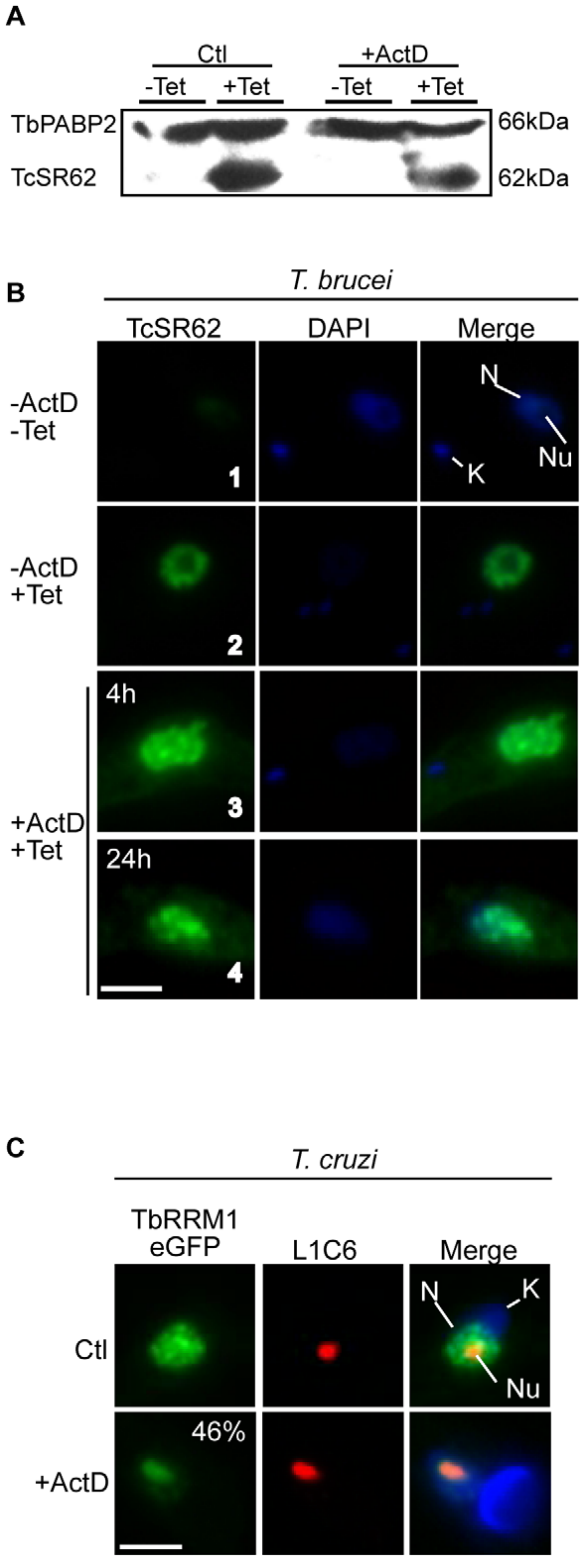
Transgene expression analysis of TcSR62 in *T. brucei* and TbRRM1 in *T. cruzi* demonstrated that the pathway/mechanism involved in nucleolar relocalization of RBPs is absent in *T. brucei*. (A) Western blot showing expression of TcSR62 in *T. brucei* after 24 h of induction with Tet 1 µg/ml in parasites untreated or subjected to ActD for 4 h. TbPABP2 was included as loading control. (B) Immunofluorescence images for TcSR62 expressed (green) in *T. brucei* after 24 h of Tet-induction subjected or not to ActD for 4 h or 24 h. The third column on the right represents an overlap of TcSR62 and DAPI. (C) Images for TbRRM1 (green) expressed as an eGFP-fusion in *T. cruzi* using a pTEX vector and colocalized with the nucleolar marker L1C6 (red) either before or after incubating the parasites with ActD for 24 h. The third column on the right is an overlap of TbRRM1-eGFP, L1C6 and DAPI. Nuclei were counterstained with DAPI (blue). The quantification for TbRRM1-eGFP is expressed as the mean from at least three independent experiments. N: nucleus, K: kinetoplast, Nu: nucleolus. Size bars represent 2 µm. Representative nuclei are shown.

As shown previously in *T. cruzi* [10], the RBPs TcSR62 (Tc00.1047053511621.50) and TcPABP2 (Tc00.1047053508461.140) were mobilized to the nucleolus in response to ActD treatment. The unexpected results that their orthologues in *T. brucei* (TbRRM1 and TbPABP2, respectively) did not accumulate into the nucleolus in response to this treatment (Figure 1C and D) might be explained in at least two possible ways: i) both orthologues in *T. brucei* lack functional nucleolar signals, which seems quite unlikely, in fact, sequence alignment analysis between TcSR62 and TbRRM1 showed that the same structural domains and sequence elements are present in both proteins (Figure S2); or ii) the mechanism/pathway behind nucleolar relocalization of RBPs is not operational in *T. brucei*. If the latter hypothesis is correct, we would then expect that TcSR62, expressed in this parasite, could not be accumulated into the nucleolus in response to ActD treatment. To test this, we expressed a TcSR62 transgene in *T. brucei* procyclic parasites using a Tetracycline (Tet)-inducible vector. We first confirmed the expression of TcSR62 by Western blot (Figure 2A) and then analyzed its behaviour under ActD treatment by immunofluorescence. In non-induced parasites, the antiserum against TcSR62 barely detected the endogenous TbRRM1 (Figure 2B, panel 1). However, after 24 h of Tet-induction, TcSR62 was detected mainly in nuclear speckled-like structures (Figure 2B, panel 2), being excluded from the nucleolus. When parasites were induced with Tet for 24 h and then subjected to ActD treatment for 4 h (Figure 2B, panel 3), instead of showing nucleolar accumulation, TcSR62 remained in more rounded speckles, which appeared coalesced all over the nucleus, displaying a pattern similar to that of TbRRM1 (compare with Figure 1C). Similar results were observed after 24 h of ActD treatment (Figure 2B, panel 4). As this result suggested that the mechanism was not operational in *T. brucei*, we then thought that TbRRM1 should be able to mobilize to the nucleolus if expressed in a *T. cruzi* background. To test this idea, we expressed a transgenic TbRRM1 as a C-terminal eGFP fusion protein using the pTEX vector [29]. Under normal conditions, it showed a nuclear speckled-like pattern as in *T. brucei* (Figure 2C, top panels). However, when parasites were subjected to ActD, TbRRM1 was accumulated into the nucleolus in 46% of epimastigote parasites (Figure 2C, bottom panels).

The mobilization of TbRRM1 to the nucleolus when expressed in *T. cruzi* clearly shows that TbRRM1 contains the necessary sequence elements to be targeted to the nucleolus. On the other hand, the lack of TcSR62 nucleolar transport in *T. brucei* reinforces our initial idea that the mechanism/pathway that transports RBPs to the nucleolus is missing in *T. brucei*.

## Discussion

Recently, the resolution of nucleolar proteomes in several organisms has provided insights into the role of the nucleolus in numerous cellular processes [20]–[22]. For instance, these projects have unexpectedly shown the nucleolar presence of RBPs required in different steps of mRNA metabolism. In this frame, we have recently found in *T. cruzi* that a subset of RBPs, involved in mRNA metabolism, is accumulated into the nucleolus in response to ActD treatment [10]. These results, prompted us to evaluate whether this mechanism/pathway could also be present in other members of the trypanosomatid lineage. Interestingly, we found that the RBP LmxPABP2 from *L. mexicana* and a transgenic RBP from *T. cruzi* (TcPTB2) are accumulated into the nucleolus in response to long-term ActD treatment (Figure 1A, B and S1A), suggesting that this mechanism is also present in other trypanosomatids. However, a different picture was seen in *T. brucei*. In this parasite, we focused our studies on two RBPs related to the mRNA metabolism: a nuclear one (TbRRM1) and a cytoplasmic one (TbPABP2). To our surprise, neither protein was relocalized to the nucleolus when the parasites were incubated in the presence of ActD, even when incubated for 24 h. In fact, TbRRM1 behaved more similarly to SR proteins from plants or mammals, being accumulated in more rounded nuclear speckles-like structures [30]–[32]. The presence of nucleolar accumulation of RBPs in *T. cruzi* and *L. mexicana* but not in *T. brucei* was unexpected, since these parasites belong to the trypanosomatid family. Nevertheless, it should be noted that among these organisms, significant differences in molecular mechanisms have also been found, being the RNAi mechanism the most remarkable case [33]. This post-transcriptional mechanism, which is well conserved through the evolution of eukaryotes, including *T. brucei*, is nonfunctional in both *T. cruzi* and *L. mexicana* [34], [35].

To further demonstrate that the mechanism behind nucleolar relocalization of RBPs might be absent in *T. brucei*, we expressed TcSR62 (from *T. cruzi*) in *T. brucei* parasites and vice versa, TbRRM1 (from *T. brucei*) in *T. cruzi* epimastigotes (it is worth mentioning that both proteins are orthologues). As expected, TcSR62 did not accumulate into the *T. brucei* nucleolus in response to ActD, behaving as the endogenous TbRRM1 (Figure 2B). On the other hand, TbRRM1 was able to relocate to the nucleolus of *T. cruzi* under the same treatment (Figure 2C), suggesting that molecular determinants for nucleolar translocation are present in its sequence. Taken together, all these results strongly suggest that the mechanism involved in the nucleolar relocalization of RBPs is absent in *T. brucei*. One plausible explanation is that *T. brucei* has lost one or more key unidentified components which might be required to allow nucleolar relocalization of RBPs. This possibility has a precedent, since, as it has been previously reported, neither AGO1 homologues nor any other gene required to elicit the RNAi mechanism are present in *T. cruzi* and *L. mexicana*, where this pathway has been lost [33]–[35].

Finally, our results suggest that the mechanism driving RBPs nucleolar relocalization seems to have been lost/retained by different members of the trypanosomatid family during the evolution of this particular group of organisms.

## Materials and Methods

### Trypanosomes and reagents

*T. cruzi* CL Brener epimastigotes were cultured in BHT medium containing brain heart infusion, 0.3% tryptose, 0.002% bovine hemin and 10% heat-inactivated fetal calf serum (BHT 10%). *L. mexicana* promastigotes (Costa Rica strain) were cultured in BHT 20%. *T. brucei* procyclic parasites (29–13 strain) were cultured in SDM79 medium supplemented with 10% heat-inactivated fetal calf serum, 50 µg/ml of hygromycin B and 15 µg/ml of geneticin. *T. brucei* parasites expressing TcSR62 were also supplemented with 3 µg/ml of phleomycin. Inductions were performed incubating transfected parasites with 1 µg/ml of Tet for 24 h. Parasite cultures were taken in a late logarithmic growth phase at a cell density of 2.5–3.5×10^7^/ml parasites for *T. cruzi* and *L. mexicana* and 0.5×10^7^/ml parasites for *T. brucei*.

*T. cruzi* and *L. mexicana* were treated with ActD for 24 h. *T. brucei* parasites were incubated with ActD either for 4 h or 24 h. ActD was used at a final concentration of 50 µg/ml (Sigma).

### Protein Extract

For total extract preparation, parasites were resuspended in lysis buffer (10 mM Tris-HCl (pH 7.6), 150 mM NaCl, 1 mM, 50 µM E64 (trans-epoxy succinyl amido (4-guanidino), phenylmethylsulfonyl fluoride, 1 mM and 0.5% Nonidet P-40) and incubated on ice for 15 min and then mixed with one volume of reducing cracking buffer 2×.

### Western Blotting

Western blot was performed as recently described [10]. The primary antibodies used were polyclonal anti-TcSR62 (1∶1000) and polyclonal anti-TcPABP2 (1∶1000). The secondary antibody used was horseradish peroxidase-conjugated goat (1∶4000), developed with the Supersignal® West Pico Chemiluminescent Substrate (Pierce) according to the manufacturer's instructions.

### Immunofluorescence

Immunofluorescences were performed as recently described [10]. The primary antibodies were monoclonal (L1C6, 1∶200), polyclonal anti-TcSR62 (1∶6000), polyclonal anti-TcPABP2 (1∶1000) and polyclonal anti-TbRRM1 (1∶1000). Secondary goat anti-rabbit or anti-mouse antibodies AlexaFluor 488 or AlexaFluor 594 (Molecular Probes) were used at 1∶1000 dilutions. Finally, cells were mounted in 1 µg/ml DAPI prepared in Fluorsave (Calbiochem). Analysis of subcellular localization was performed in a Nikon Eclipse E600 microscope coupled to a SPOT RT colour camera (Diagnostic Instruments). Merged images were obtained by superimposing the indicated image files in SPOT Software 4.0.9 (Diagnostic Instruments).

### GFP fusion construct

Full-length TbRRM1 and TcPTB2 were amplified by PCR using the primers listed below and cloned, the former into the BamHI site and the latter into the EcoRI and HindIII sites of pTEX-eGFP kindly provided by Dr. J.M. Kelly [29].

TbRRM1

UTbRRM1_BamHI: GGATCCATGCAACAATATACCCTTCG

Rv_TbRRM1_NoSTOP_BamHI: CGGGATCCCGGTCCCTTACGCGGTC

TcPTB2

PTB_exp1: CCGAATTCATGATGTCCGTGGTCTTGC

Rv_PTB_NoSTOP_HindIII: AAGCTTCCCTCCTCTTCAGTTGGT

### Parasite Transfections

*T. cruzi* transfections were carried out as recently described [10]. For *L. mexicana*, transfections were carried out with a BTX 600 electroporator in a 4-mm gap cuvette. A total of 100×10^6^ parasites were harvested, washed twice in cold PBS, once in cold electroporation buffer (137 mM NaCl, 20 mM HEPES, 6 mM glucose, 5 mM KCl, 0.7 mM NaH_2_PO_4_) and resuspended in 0.4 ml of electroporation buffer with 50 µg of supercoiled plasmid DNA. The electroporation setting was: 1400 microfarads, 335 V, and 24 Ω. Parasites were recovered in 10 ml of BHT supplemented with 20% fetal calf serum (Natocor) and 36 h later geneticin (Sigma) was added at a final concentration of 50 µg/ml.

## Supporting Information

Figure S1 **Effects of ActD treatment on the localization of PABP2 and TbRRM1 showing whole parasites.** Immunofluorescence images of the corresponding protein in ActD-treated and untreated parasites. (A) LmxPABP2 (green) was colocalized with the nucleolar marker L1C6 (red) in *L. mexicana*. Nuclei were counterstained with DAPI (blue). (B) Immunofluorescence images for TbRRM1 (green) and TbPABP2 (red) in *T. brucei*. Size bars represent 2 µm. Representative parasites are shown.
(TIF)

Figure S2 **Sequence alignment between TbRRM1 and TcSR62.** The RRMs (black), zinc finger (red), arginine rich (brown) and RS (pink) domains as well as the NLS (blue) element are indicated. The numeration is referred to TbRRM1. Within aligned regions, identical amino acids are shown in red letters over yellow background, while similar amino acids are shown with a green background. When necessary, spaces were inserted within the sequences to allow better alignment (indicated with slash lines). Sequence alignment analysis was performed by using Vector NTI software, whereas domains were assigned by using the Prosite database.
(TIF)
