# Supplementary figures and images for: Nucleolar Accumulation of RNA Binding Proteins Induced by ActinomycinD Is Functional in Trypanosoma cruzi and Leishmania mexicana but Not in T. brucei
Source: PLoS One. 2011 Aug 31;6(8):e24184. doi: 10.1371/journal.pone.0024184 (PMC3164162; doi:10.1371/journal.pone.0024184)

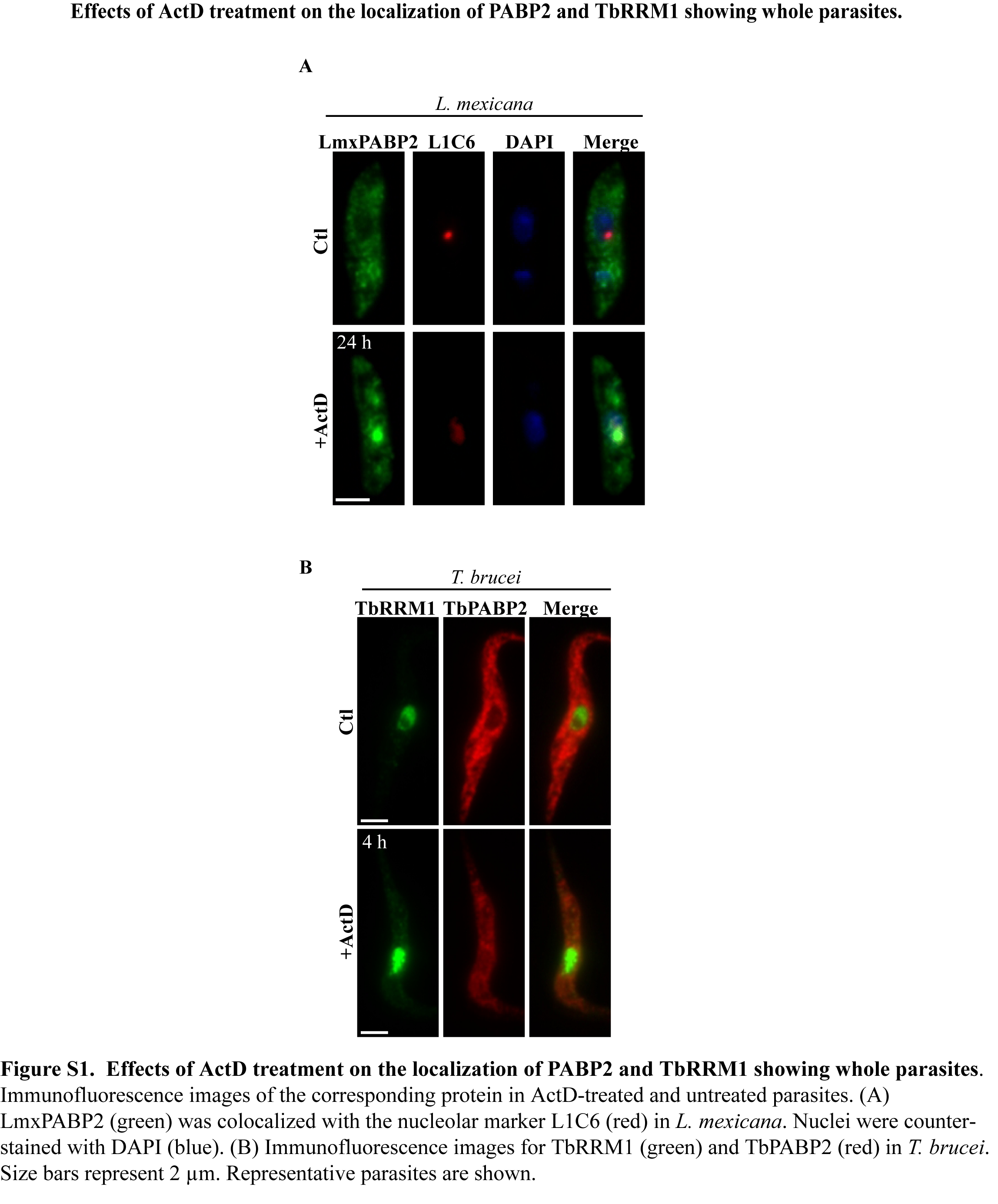

Supplement: Figure S1 — Effects of ActD treatment on the localization of PABP2 and TbRRM1 showing whole parasites. Immunofluorescence images of the corresponding protein in ActD-treated and untreated parasites. (A) LmxPABP2 (green) was colocalized with the nucleolar marker L1C6 (red) in L. mexicana. Nuclei were counterstained with DAPI (blue). (B) Immunofluorescence images for TbRRM1 (green) and TbPABP2 (red) in T. brucei. Size bars represent 2 µm. Representative parasites are shown. (TIF) [file pone.0024184.s001.tif]

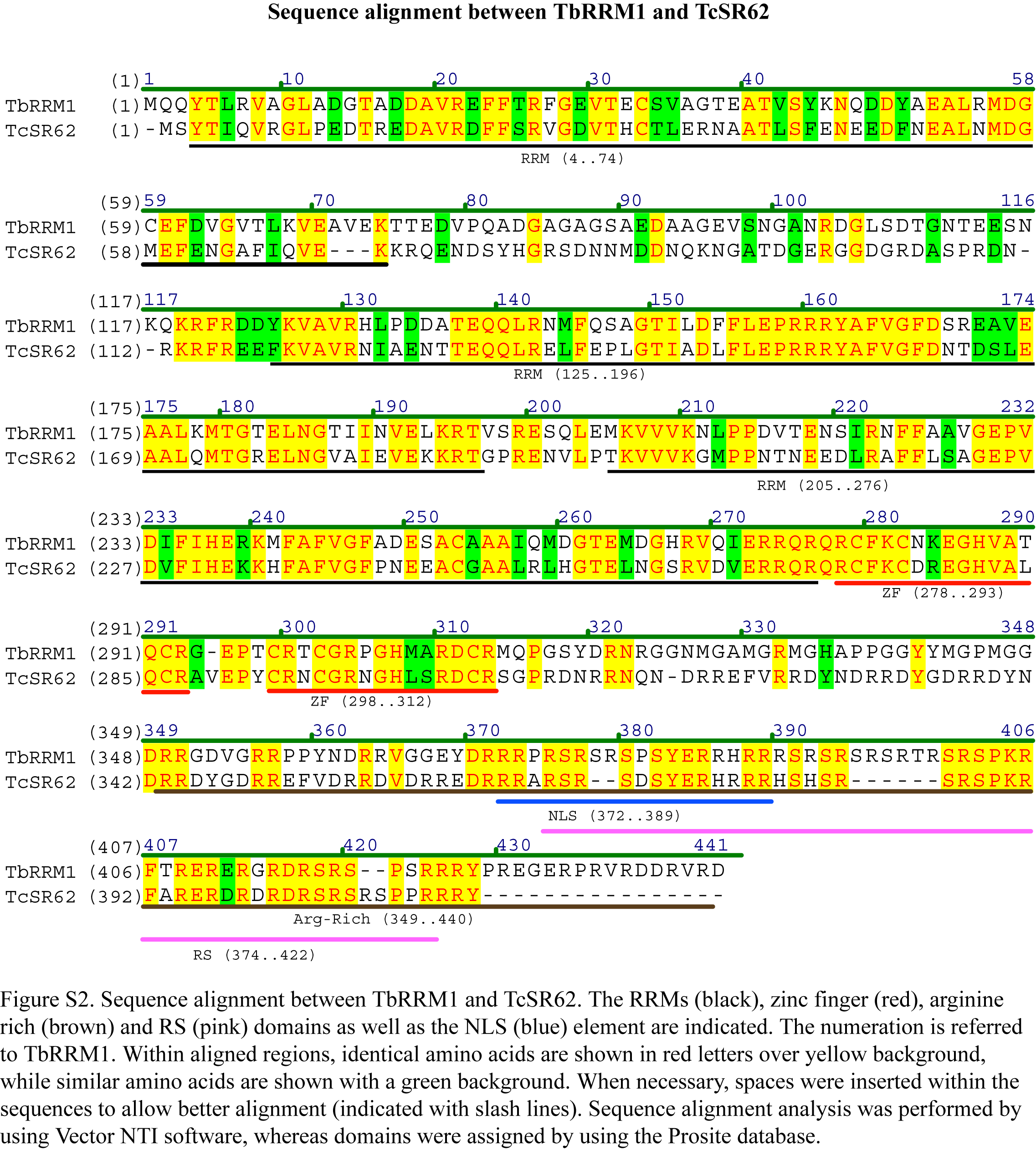

Supplement: Figure S2 — Sequence alignment between TbRRM1 and TcSR62. The RRMs (black), zinc finger (red), arginine rich (brown) and RS (pink) domains as well as the NLS (blue) element are indicated. The numeration is referred to TbRRM1. Within aligned regions, identical amino acids are shown in red letters over yellow background, while similar amino acids are shown with a green background. When necessary, spaces were inserted within the sequences to allow better alignment (indicated with slash lines). Sequence alignment analysis was performed by using Vector NTI software, whereas domains were assigned by using the Prosite database. (TIF) [file pone.0024184.s002.tif]
